# Integrated Analysis of the Expression Characteristics, Prognostic Value, and Immune Characteristics of PPARG in Breast Cancer

**DOI:** 10.3389/fgene.2021.737656

**Published:** 2021-09-09

**Authors:** Jianbin Wu, Mingmin Luo, Zhuangwei Chen, Lei Li, Xiaoxi Huang

**Affiliations:** ^1^Department of Breast, Fujian Maternity and Child Health Hospital, Affiliated Hospital of Fujian Medical University, Fuzhou, China; ^2^Reproductive Medicine Center, Fujian Maternity and Child Health Hospital, Affiliated Hospital of Fujian Medical University, Fuzhou, China; ^3^Department of Pathology, University of Otago, Dunedin, New Zealand

**Keywords:** breast cancer, PPARG, biomarker, TCGA, GEO

## Abstract

**Background:** Breast cancer (BRCA) is the most frequent malignancy. Identification of potential biomarkers could help to better understand and combat the disease at early stages.

**Methods:** We selected the overlapping genes of differential expressed genes and genes in BRCA-highly correlated modules by Weighted Gene Co-Expression Network Analysis (WGCNA) in TCGA and GEO data and performed KEGG and GO enrichment. PPARG was achieved from Protein-Protein Interaction (PPI) network analysis and prognostic analysis. TIMER, UALCAN, GEO, TCGA, and western blot analysis were used to validate the expression of PPARG in BRCA. PPARG was further analyzed by DNA methylation, immune parameters, and tumor mutation burden.

**Results:** Among 381 overlapping genes, the lipid metabolic process was identified as highly enriched pathways in BRCA by TCGA and GEO data. When the prognostic analysis of 10 core genes by PPI network was performed, results revealed that high expression of PPARG was significantly correlated to a better prognosis. PPARG was lesser expression in BRCA according to TIMER, UALCAN, GEO, TCGA, and western blot in both mRNA level and protein level. PPARG had several high DNA methylation level sites and the methylation level is negatively correlated to expression. PPARG is also correlated to TNM stages, tumor microenvironment, and tumor burden.

**Conclusions:** Findings of our study identified the PPARG as a potential biomarker by confirming its low expression in BRCA and its correlation to prognosis. Moreover, its correlation to DNA methylation and tumor microenvironment may guide new therapeutic strategies for BRCA patients.

## Introduction

Breast cancer (BRCA) is one of the most common, complex and aggressive malignant tumors among females and it is the 2nd most occurring and 5th major cause of cancer related deaths in women all over the world (Kotsopoulos, 2018; Guo et al., 2020; t’Kint de Roodenbeke et al., 2020; Yu et al., 2021). Despite the huge advances in early screening and various therapies for BRCA patients in the last decades, their prognosis remains poor (Lang et al., 2017; Guney Eskiler et al., 2018; Mylavarapu et al., 2018). Therefore, there is an urgent need to explore new and effective biomarkers and therapeutic targets to improve the prognosis and life quality of BRCA patients.

Peroxisome Proliferator Activated Receptor Gamma (PPARG) is a member of the peroxisome proliferator-activated receptor (PPAR) subfamily of nuclear receptors and it is encoded by PPARG gene in humans (González-Castro et al., 2018; Ghallab and Seddek, 2020; Lian et al., 2020). PPARs (PPAR-alpha, PPAR-delta, and PPAR-gamma) can heterodimerize with retinoid X receptors (RXRs) and then directly regulate gene transcription. Although the main role of PPAR-gamma is to regulate adipocyte differentiation, dysregulation of PPAR-gamma has been the cause for numerous deadly diseases like diabetes and cancer. Furthermore, PPARG possess high affinity with the thiazolidinediones (TZDs) class of antidiabetic drugs, supporting its potential role for cancer therapy (Liu et al., 2019; Wu et al., 2019; Shi et al., 2020). In breast cancer, PPARG has been demonstrated with antitumor activities but its mechanism of action still needs further research (Zhou et al., 2015; Almeida et al., 2018; Xu et al., 2019; Winkler et al., 2020; Yang et al., 2020; Wilson et al., 2021).

In the current investigation, we systematically analyzed PPARG mRNA expression signature, genetic and epigenetic characteristics, prognostic value, correlation with tumor-infiltrating immune cells, and related pathways in BRCA. We further verified our findings through the TCGA database, GEO database, western blot, and various online websites. These findings support PPARG as a potential biomarker or therapeutic target for BRCA and provide information for a better understanding of its role in BRCA.

## Materials and Methods

### Data Processing

The RNA sequencing (RNA-Seq) expression data for 1,109 BRCA samples and 113 normal tissue samples with corresponding clinical information was downloaded from the TCGA database on September 2020.^1^ Gene expression profiles and clinical traits of GSE42568 (104 BRCA samples and 17 normal tissue samples) were downloaded from the GEO database.^2^ GPL570 [HG-U133_Plus_2] Affymetrix Human Genome U133 Plus 2.0 Array was used to extract the expression profile information of the GSE42568 dataset. Through R package “limma,” the TCGA and GEO data were analyzed for significant difference genes, and the screening conditions were based on false discovery rate (FDR) < 0.05 and | log2 fold change (FC)| ≥ 1.

### Weighted Gene Co-expression Network Analysis

The weighted gene co-expression network analysis (WGCNA) was performed as described previously with some modifications. The TCGA and GEO data were used respectively for WGCNA using the “WGCNAR” R package. Weighted gene co-expression network analysis was used to explore the relationship between the tumor modules and the normal modules.

### KEGG Pathway and GO Enrichment Analysis

To assess the biological functions behind key genes the “clusterProfiler” R package was selected and gene ontology (GO), functional annotation and Kyoto Encyclopedia of Genes and Genomes (KEGG) analysis were performed (Shi et al., 2020). A *p*-value? < ?0.05 and FDR < 0.05 were considered statistically significant.

### Protein-Protein Interaction Network Analysis

The protein-protein interaction information between key genes was analyzed by using the online tool, Search Tool for the Retrieval of Interacting Genes (STRING)^3^ and disconnected nodes were hide in the network (Szklarczyk et al., 2015, 2017, 2019). The Cytoscape_v3.7.0 software was used to further construct the PPI network. In addition, we predicted the important nodes and subnets in the network through the cytoHubba plug-in.

### Gene Set Enrichment Analysis

Gene Set Enrichment Analysis was carried out using GSEA software, version 4.0.1 for the identification of signaling pathways that are differentially activated between the high PPARG expression group and low PPARG expression group.

### Estimation of Tumor Microenvironment Score

To explore the relationship between breast cancer tumor microenvironment and PPARG, we evaluated the Immune Score, and Stromal Score of each sample through the R package “ESTIMATE” (Yoshihara et al., 2013). Immune Score and Stromal Score are positively correlated with the ratio of immune, and stroma, respectively. The higher the score, the larger the ratio of corresponding components in the tumor microenvironment.

### Estimation of Immune Cell Type Fractions

To assess the differences in immune cell subtypes, we used the R package “CIBERSORT” (Chang et al., 2020; Ye et al., 2020) and examined the proportion of 22 immune cell subtypes (naive B cells, memory B cells, plasma cells, CD8 T cells, naive CD4 T cells, resting memory CD4 T cells, activated memory CD4 T cells, follicular helper T cells, T cells regulatory, gamma delta T cells, resting NK cells, activated NK cells, monocytes, macrophages M0, macrophages M1, macrophages M2, resting dendritic cells, activated dendritic cells, resting mast cells, activated mast cells, eosinophils, and neutrophils). Samples with a statistical significance difference of *p* < 0.05 were used for further analysis.

### Estimation of Tumor Mutational Burden Scores

TMB is defined as the total number of mutations per megabase in tumor tissue. To analyze the relationship between PPARG and TMB, we calculated the mutation of each sample in BRCA through the “maftools” R software package.

### Online Database Analysis: TISIDB, TIMER, UALCAN, and Kaplan-Meier Plotter

TISIDB^4^ is an integrated database website for tumor-immune system interactions (Ru et al., 2019). Therefore, we employed TISIDB to examine the expression of PPARG in different immune subtypes, including C1 (wound healing), C2 (IFN-γ dominant), C3 (inflammatory), C4 (lymphocyte deplete), C5 (immunologically quiet), and C6 (TGF-β dominant) subtypes.

TIMER^5^ is a comprehensive resource for systematical analysis of immune infiltrates across diverse cancer types (Li et al., 2016, 2017). After confirming the expression of PPARG in various tumors by the TIMER database, we studied the interaction between PPARG and immune infiltration cells in the tumor using the TIMER algorithm.

UALCAN^6^ is a comprehensive, user-friendly, and interactive web resource for analyzing cancer OMICS data (Chandrashekar et al., 2017). Thus, we used the UALCAN to examine the mRNA expression level, protein expression level, and promoter methylation level of PPARG in breast cancer.

Kaplan-Meier plotter^7^ is a comprehensive database that can quickly evaluate the gene prognosis and survival (Györffy et al., 2010; Nagy et al., 2021). We used the Kaplan–Meier plotter to investigate the prognostic value of PPARG mRNA in breast cancer.

### Cell Culture

The human normal breast cell line (Hs 578Bst) was obtained from the Cell Resource Center, Shanghai Institutes for Biological Sciences, Chinese Academy of Sciences. MCF7 cell line, UACC812 cell line, and SK-BR-3 cell line were received from the Cell Resource Center, Peking Union Medical College. Hs 578Bst cells were cultured with DMEM and supplemented with 10% FBS and 30 ng/ml EGF. MCF7 cells were cultured with DMEM and supplemented with 10% FBS. UACC812 cells and SK-BR-3 cells were all cultured with L15: Leibovitz Medium and supplemented with 10% FBS.

### Western Blot

Western blot analysis was carried out according to the procedure mentioned earlier with some amendments (Bass et al., 2017; Pillai-Kastoori et al., 2020; Li et al., 2021). Antibodies used in western blot are as follows: Anti-PPARG (cat. no. sc-7273, 1:500 dilution) was obtained from the Santa Cruz Biotechnology (Santa Cruz, CA, United States); β-actin (cat. no. A5441, 1: 50,000 dilution) was received from Sigma-Aldrich (Merck KGaA, Darmstadt, Germany); the secondary antibodies (cat. no. 7076, 1: 2,000 dilution) were obtained from Cell Signaling Technology (Beverly, MA, United States).

### Statistical Analysis

All statistical analysis were performed using the R version 4.0.3 software. The “ggplot2,” “ggforest,” “maftools,” “cowplot,” “VennDiagram,” and “ggplotify” packages in R were used for visualization of analysis results. Based on three independent experiments, data were expressed as mean ± SEM. A *p*-value of < 0.05 was considered as statistically significant.

## Results

### Screening of Breast Cancer-Related Genes

**FIGURE 1:**
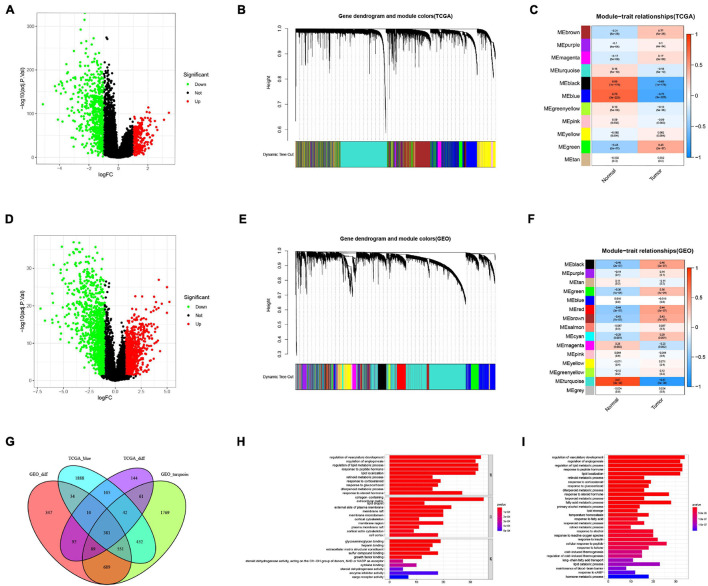
Screening of breast cancer-related genes. **(A)** Identification of DEGs in TCGA data. **(B)** A dendrogram of the differentially expressed genes clustered based on different metrics in TCGA data. Each branch in the figure represents one gene, and every color below represents one co-expression module. **(C)** Heatmap of the correlation between the module eigengenes and BRCA in TCGA data. **(D)** Identification of DEGs in GEO data. **(E)** A dendrogram of the differentially expressed genes clustered based on different metrics in GEO data. **(F)** Heatmap of the correlation between the module eigengenes and BRCA in GEO data. **(G)** Venn diagram showed that there were 381 overlapping genes. **(H)** GO enrichment analysis. **(I)** KEGG enrichment analysis.

We first investigated the genes related to BRCA by conducting a differential gene (DEG) analysis on the TCGA data (Figure 1A) and GEO data (Figure 1D), and then we identified the modules related to BRCA in the TCGA data (Figures 1B,C) and GEO data (Figures 1E,F) through the WGCNA algorithm. The results revealed that the module MEblue presented highest correlation with BRCA in the TCGA data, and the module MEturquoise displayed the highest correlation with BRCA in the GEO data. At the intersection of these DEGs and modular genes, the results confirmed that there were 381 overlapping genes (Figure 1G).

In addition, we performed GO and KEGG enrichment analysis on these genes. The GO results (Figure 1H) indicated that genes were significantly enriched in the regulation of vasculature development, regulation of angiogenesis, regulation of lipid metabolic process, response to peptide hormone, lipid localization, retinoid metabolic process, response to corticosteroid, response to glucocorticoid, diterpenoid metabolic process, response to steroid hormone, collagen-containing extracellular matrix, lipid droplet, external side of plasma membrane, membrane raft, membrane microdomain, cortical cytoskeleton, membrane region, plasma membrane raft, cortical actin cytoskeleton, cell cortex, glycosaminoglycan binding, heparin binding, extracellular matrix structural constituent, sulfur compound binding, growth factor binding, steroid dehydrogenase activity, acting on the CH-OH group of donors, NAD or NADP as acceptor, cytokine binding, steroid dehydrogenase activity, enzyme inhibitor activity, and cargo receptor activity. The findings from KEGG analysis (Figure 1I) demonstrated that genes were mainly enriched in regulation of vasculature development, maintenance of blood-brain barrier, retinol metabolic process, long-chain fatty acid transport, response to cAMP, response to fatty acid, lipid storage, primary alcohol metabolic process, regulation of cold-induced thermogenesis, cold-induced thermogenesis, isoprenoid metabolic process, terpenoid metabolic process, diterpenoid metabolic process, retinoid metabolic process, hormone metabolic process, response to ketone, temperature homeostasis, response to glucocorticoid, response to corticosteroid, response to reactive oxygen species, response to alcohol, response to insulin, lipid catabolic process, cellular response to peptide, response to a steroid hormone, fatty acid metabolic process, lipid localization, regulation of angiogenesis, response to peptide hormone, and regulation of lipid metabolic process.

### Identification of the Key Genes

**FIGURE 2:**
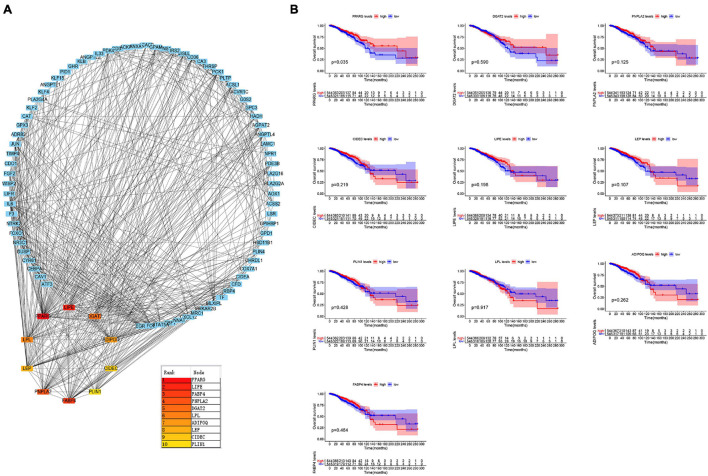
Identification of the key genes. **(A)** Protein-Protein Interaction network analysis and prediction of its core genes. **(B)** Kaplan–Meier survival curves were generated for selected 10 genes extracted from the comparison of groups of high (red line) and low (blue line) gene expression.

Protein-protein interaction network analysis is of great significance for understanding protein functions and relationships. To further explore the key genes in breast cancer, we performed the PPI network analysis on these 381 genes through the STRING website and visualized them through Cytoscape software (Figure 2A). CytoHubba is a plug-in of Cytoscape software. CytoHubba provides 11 topological analysis methods, which can help us measure nodes by their network features to infer their importance in the network, and it can identify central elements of biological networks (Chin et al., 2014; Li et al., 2020). We predicted the core genes in the PPI network through the cytoHubba plug-in, and analyzed the prognosis of these genes (Figure 2B). The outcomes revealed that high expression levels of PPARG were associated with a better prognosis. These results are in accordance with the findings of the Kaplan-Meier plotter website (Supplementary Figure 1).

### The Expression Level of PPARG in BRCA

**FIGURE 3:**
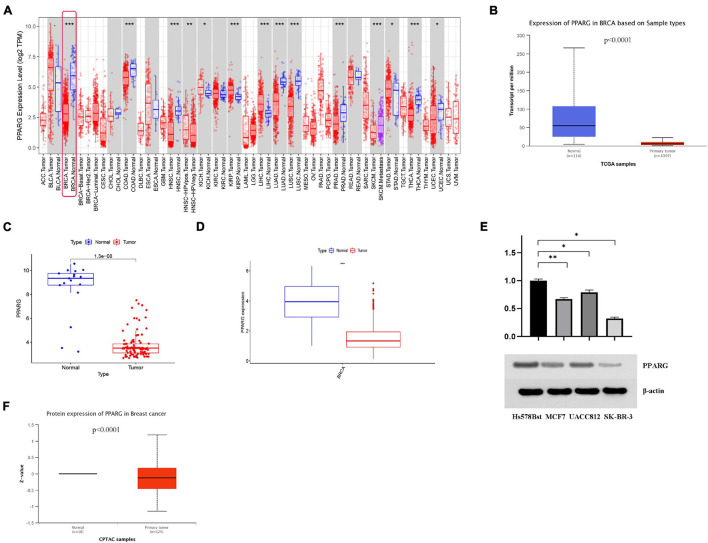
The expression level of PPARG in BRCA. **(A)** The TIMER database was used to evaluate the mRNA expression of PPARG. **(B)** The UALCAN database was used to evaluate the mRNA expression of PPARG. **(C)** The mRNA expression of PPARG was assessed using data from GEO. **(D)** The mRNA expression of PPARG was assessed using data from TCGA. **(E)** Western blot analysis results showed that compared with normal breast cell line (Hs 578Bst), the expression of PPARG in BRCA cell line (MCF7, UACC812, and SK-BR-3) was significantly reduced. **(F)** The UALCAN database was used to evaluate the protein expression of PPARG. *P < 0.05, ** *P* < 0.01, *** *P* < 0.001.

The findings from the TIMER database expressed that the mRNA expression level of PPARG in the normal group was significantly higher than that in the tumor group (Figure 3A). We further verified it with TCGA data (Figure 3D), GEO data (Figure 3C), and UALCAN data (Figure 3B). In addition, we analyzed the protein expression level of PPARG in BRCA. The outcomes from Western blot analysis (Figure 3E) and UALCAN analysis (Figure 3F) exhibited that PPARG was under-expressed in the tumor group. In summary, we can conclude that the expression level of PPARG in the normal group was significantly higher than that in the tumor group.

### DNA Methylation Analysis of PPARG in BRCA

**FIGURE 4:**
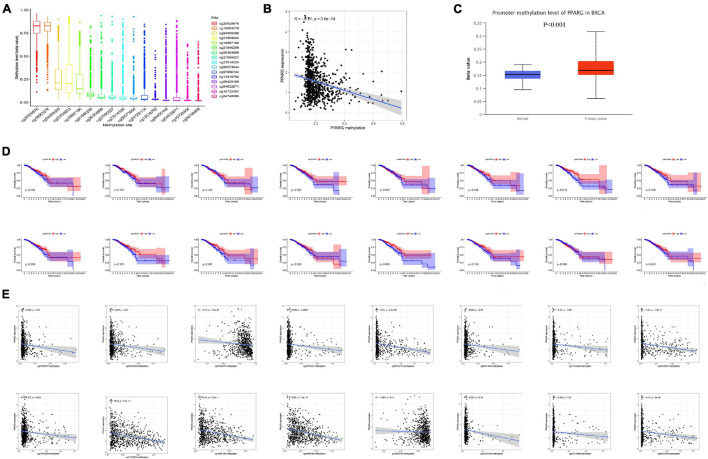
DNA methylation analysis of PPARG in BRCA. **(A)** Boxplot of methylation sites. **(B)** Correlation between PPARG methylation level and mRNA expression level. **(C)** The methylation expression level of PPARG was accessed from UALCAN. **(D)** The correlation between the level of site methylation and the prognosis. **(E)** Correlation between site methylation level and mRNA expression level.

DNA Methylation is an important epigenetic modification, which has an important regulatory effect on gene expression. Thus, we conducted DNA methylation analysis on PPARG in BRCA. We studied the methylation sites of PPARG (Figure 4A) as well as correlation between the methylation level and prognosis (Figure 4D). The results showed that high methylation levels of cg08573844 (*p* = 0.010), cg25929976 (*p* = 0.005), and cg27095527 (*p* = 0.045) were significantly related to better prognosis. We also found that the methylation level of PPARG has a negative correlation with the expression level of PPARG (Figure 4B), and the methylation level of PPARG in the tumor group was significantly higher than that in the normal group (Figure 4C). Further, we analyzed the correlation between site methylation and PPARG expression, and the outcomes demonstrated that most site methylation has a negative correlation with PPARG expression (Figure 4E). Therefore, the abnormal decrease in PPARG mRNA expression in BRCA may be the result of higher DNA methylation levels.

### Correlation Analysis Between PPARG and Clinical

**FIGURE 5:**
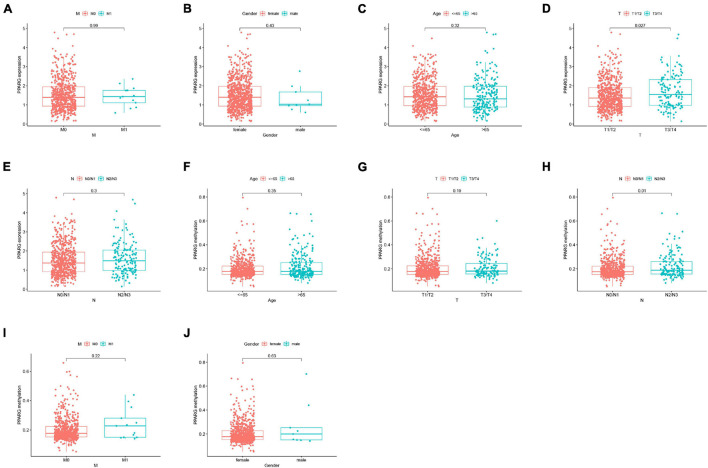
Correlation analysis between PPARG and clinical. **(A–E)** Correlation analysis between PPARG mRNA expression level and clinical. **(F–J)** Correlation analysis between PPARG methylation level and clinical.

We further analyzed the correlation between the expression level of PPARG mRNA and the clinical (Figures 5A–E) and the correlation between the methylation level of PPARG and the clinical (Figures 5F–J). The findings from the analysis of the correlation between PPARG and clinical revealed that the T stage is significantly correlated with the mRNA expression of PPARG (Figure 5D), and the N stage is significantly correlated with the methylation level of PPARG (Figure 5H). The patient’s clinical information is displayed in Supplementary Table 1.

### Relationship Between PPARG and the Tumor-Immune Microenvironment

**FIGURE 6:**
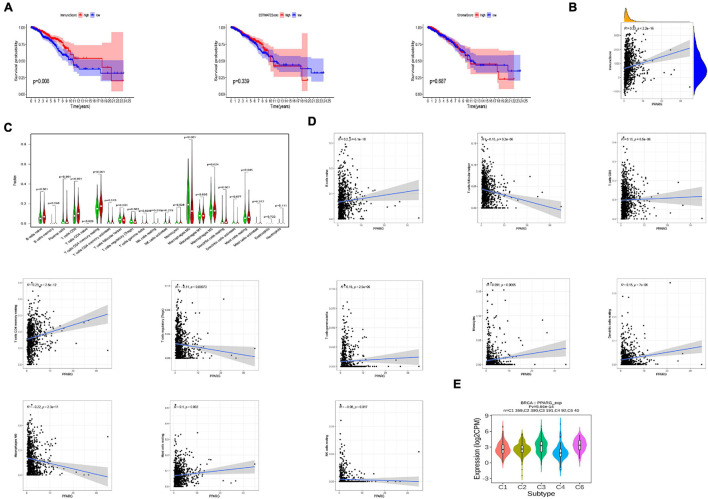
Relationship between PPARG and the tumor-immune microenvironment. **(A)** Evaluate the immune microenvironment of TCGA-BRCA through the ESTIMATE algorithm. **(B)** Correlation analysis between PPARG expression level and immune score. **(C)** Evaluate the level of immune cells between the high- (red) and low- (green) PPARG expression groups through the CIBERSORT algorithm. **(D)** Correlation analysis between PPARG and immune cells. **(E)** The expression of PPARG of different immune subtypes in BRCA was evaluated by TISIDB.

To further analyzed the PPARG in the BRCA immune microenvironment, we first evaluated the immune scores of TCGA patients through the R package “ESTIMATE” (Figure 6A). The discovery concluded that high immune scores were significantly related to a better prognosis. Another important finding was the positive correlation of PPARG expression level with the immune score (Figure 6B). In addition, we divided TCGA patients into high and low groups on the basis of mRNA expression level of PPARG. The level of immune cells between the high and low groups was examined by employing CIBERSORT algorithm (Figure 6C). The results of correlation analysis displayed that PPARG expression was positively correlated with B cells naive and T cells CD4 memory resting. PPARG expression disclosed a negative correlation with Macrophages MO (Figure 6D). We analyzed the expression levels of PPARG in different immune subtypes through the TISIDB website, and we observed significant differences in the expression characteristics of PPARG in C1, C2, C3, C4, C5, and C6 (Figure 6E).

### Prognostic Analysis of PPARG Expressions in BRCA Based on Immune Cells

**FIGURE 7:**
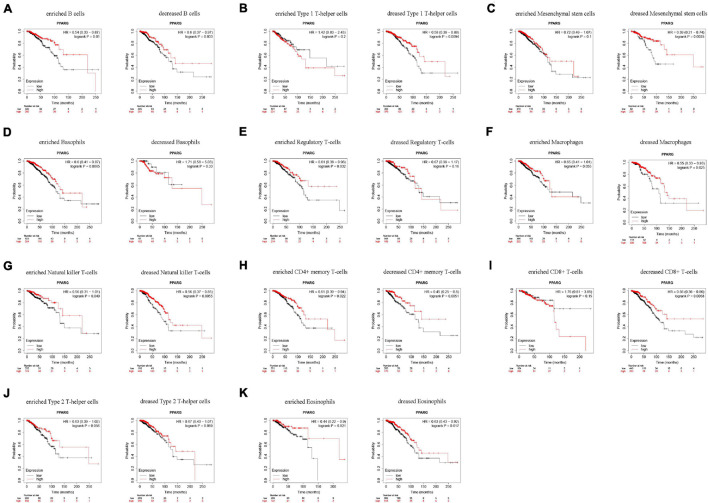
Comparison of Kaplan-Meier survival curves of the high and low expression of PPARG in BRCA based on immune cells subgroups. **(A–K)** Relationships between PPARG of different immune cells subgroup and prognoses in BRCA.

Based on the above finings, we speculated that the expressions of PPARG in BRCA affected the prognosis partly because of immune infiltration. Therefore, we did a prognosis analysis based on the expression levels of PPARG in related immune cells subgroup *via* the Kaplan Meier plotter (Figure 7). The findings demonstrated that the high expression of PPARG of BRCA in enriched B cells (*HR* = 0.54), decreased B cells (*HR* = 0.6), decreased Type 1 T-helper cells (*HR* = 0.58), decreased Mesenchymal stem cells (*HR* = 0.39), enriched Basophils (*HR* = 0.6), enriched Regulatory T-cells (*HR* = 0.61), decreased Macrophages (*HR* = 0.55), enriched Natural killer T-cells (*HR* = 0.56), decreased Natural killer T-cells (*HR* = 0.56), enriched CD4 + memory T-cells (*HR* = 0.61), decreased CD4 + memory T-cells (*HR* = 0.45), decreased CD8 + T-cells (*HR* = 0.56), enriched Eosinophils (*HR* = 0.44), and decreased Eosinophils (*HR* = 0.63) cohort performed better prognosis, respectively. These conclusions suggested that high PPARG expressions in BRCA may affect prognosis in part due to immune infiltration.

### The Relationship Between PPARG and the Immune Checkpoints

**FIGURE 8:**
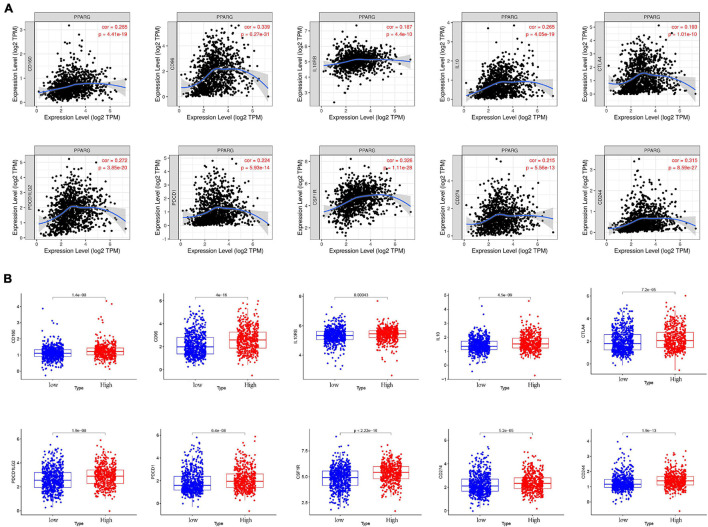
The relationship between PPARG and immune checkpoints. **(A)** Analyze the correlation between PPARG and immune checkpoints through the TIMER database. **(B)** Analysis of the difference of immune checkpoints between high- and low- PPARG expression groups.

At present, immunotherapy has played a fascinating role in tumor treatment, and immune checkpoint blockade is a brilliant method. Therefore, we explored the relationship between PPARG and immune checkpoints (Figure 8A). Through the TIMER database, we found that there is a positive correlation between PPARG and most of the immune checkpoints (CD160, CD96, IL10RB, IL10, CTLA4, PDCD1LG2, PDCD1, CSF1R, CD274, and CD244). Then we divided the TCGA patients into high and low groups based on the expression level of PPARG. We found that the high PPARG expression group had a higher level of immune checkpoint expression (Figure 8B). These findings are consistent with our previous results.

### The Relationship Between PPARG and Tumor Mutation Burden

**FIGURE 9:**
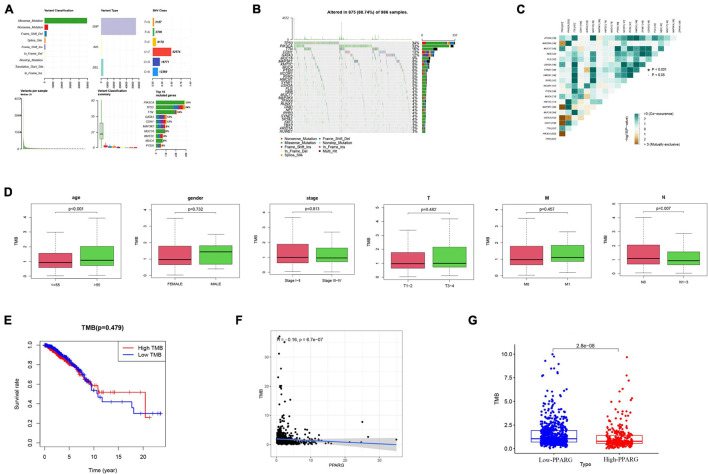
The relationship between PPARG and tumor mutation burden. **(A)** Overview of TCGA-BRCA cohort mutations. **(B)** Waterfall of the top 20 mutated genes in the TCGA-BRCA cohort. **(C)** The correlation between 20 genes. **(D)** The clinical correlation analysis. **(E)** Correlation analysis of TMB and prognosis. **(F)** Correlation analysis between PPARG and TMB. **(G)** Comparison of TMB levels in high- and low- PPARG groups.

In recent years, numerous pieces of research have been conducted on TMB. TMB is closely related to tumor progression and has the potential to become a biomarker. At this stage, we evaluated the mutation in the TCGA-BRCA cohort through the “maftools” algorithm (Figure 9A), visualized the top 20 mutated genes (Figure 9B), and the correlation between these 20 genes (Figure 9C). The results of clinical correlation analysis showed that both the >55 age group (*P* < 0.001) and the NO stage group (*P* = 0.007) have significant correlations with high TMB (Figure 9D). It was worth noting that there is no significant correlation between TMB (High or Low) and survival rate (Figure 9E). Another important finding is that PPARG is negatively correlated with TMB levels (Figure 9F), and the low PPARG group has higher TMB levels (Figure 9G).

### GSEA Analysis Related to PPARG

GSEA software was used to determine the signal pathways that are differentially activated between the high PPARG expression group and the low PPARG expression group. We visualized the first 10 related pathways (Table 1). The KEGG CALCIUM SIGNALING PATHWAY, KEGG FOCAL ADHESION, KEGG VASCULAR SMOOTH MUSCLE CONTRACTION, KEGG GLYCEROLIPID METABOLISM, and KEGG DILATED CARDIOMYOPATHY were enriched in the PPARG high expression group, and the KEGG DNA REPLICATION, KEGG PROTEASOME, KEGG OXIDATIVE PHOSPHORYLATION, KEGG SPLICEOSOME, and KEGG PROTEIN EXPORT pathways were enriched in the PPARG low expression group (Supplementary Figure 2).

**TABLE 1:** GSEA result.

**Name**	**ES**	**NES**	**NOM *P*-value**	**FDR *q*-value**
KEGG_CALCIUM_SIGNALING_PATHWAY	0.613612	2.364593	0	0.011054
KEGG_FOCAL_ADHESION	0.648454	2.363956	0	0.006037
KEGG_VASCULAR_SMOOTH_MUSCLE_CONTRACTION	0.621201	2.322039	0	0.009466
KEGG_GLYCEROLIPID_METABOLISM	0.638116	2.301838	0	0.007872
KEGG_DILATED_CARDIOMYOPATHY	0.646264	2.261655	0	0.009667
KEGG_DNA_REPLICATION	–0.81147	–2.1485	0	0.041833
KEGG_PROTEASOME	–0.68258	–2.09812	0.014113	0.034522
KEGG_OXIDATIVE_PHOSPHORYLATION	–0.59907	–1.99621	0.007797	0.043872
KEGG_SPLICEOSOME	–0.55225	–1.98048	0.010121	0.045697
KEGG_PROTEIN_EXPORT	–0.67294	–1.92061	0.011742	0.05709

*NOM *P* < 0.05 has statistical significance.*

## Discussion

Until today, BRCA is the most frequent malignancy in the world (Karami and Mehdipour, 2013; Balic et al., 2019; Harbeck et al., 2019). Although the heterogeneity at molecular level guides the treatments in BRCA, some features like locoregional tumor burden or metastatic patterns have similar impacts on therapies in all BRCA patients. BRCA is considered curable at early stages, but metastatic breast cancer with current clinical treatments has a poor prognosis (Early Breast Cancer Trialists’ Collaborative Group [EBCTCG], 2019; Im et al., 2019; Rugo et al., 2019; von Minckwitz et al., 2019; Mittendorf et al., 2020). Thus, sensitive novel prognostic biomarkers or therapeutic targets for BRCA are still in urgent need.

The current investigation is an effort to find out potential biomarkers for BRCA. Firstly, we used TCGA and GEO databases to make a cross validation. We carried out differential expressed analysis and WGCNA and those overlapping genes were considered as more potential biomarkers for further analysis study. The enrichment analysis in TCGA and GEO are almost consistent but these top pathways did not include common cancer-related pathways. To get the key genes, we performed the PPI network and Kaplan-Meier on the core genes. The results from these analysis clearly depicted that only PPARG is associated with the prognosis of BRCA patients—patients with high expression of PPARG are likely to have a better prognosis.

PPARG belongs to the nuclear receptor super family PPARs, activated by fatty acid and lipid metabolites (Saez et al., 2003). This finding is consistent with the lipid metabolic process ranked top 3 in the KEGG and GO enrichment, implying PPARG, as well as lipid metabolic process, might work as a crucial part in BRCA development. Although the main role of PPARG is to focus on adipocyte differentiation and diabetes, many pieces of research have also demonstrated that PPARG has an important impact on the growth of various cancers (Goldstein et al., 2017; Galbraith et al., 2018; Lin et al., 2019; Zou et al., 2019; Villa et al., 2020). We further provide reliable evidence that the expression level of PPARG in BRCA tumors is significantly reduced. On the one hand, TIMER, TCGA, GEO, and UALCAN displayed the lower mRNA expression of PPARG in tumor tissues than normal tissues. On the other hand, UALCAN also showed a slightly lower protein level of PPARG. Moreover, we compared the PPARG protein expression in human normal breast cell line (Hs 578Bst) and in breast cancer cell lines MCF7, UACC812, and SK-BR-3 cells to confirm the lower expressions of PPARG.

Further, we explored the PPARG’s various features in BRCA. Firstly, we noticed that the high DNA methylation levels of the three sites were significantly related to better prognosis and the high methylation level was correlated to low PPARG expression. Secondly, the DNA methylation level of PPARG was significantly higher in the tumor group as compared to the normal group. Our findings are coherent with the earlier studies and it indicates that DNA methylation might be an upstream way to regulate PPARG during BRCA progression. According to clinical correlation study, PPRAG expression in T1/T2 was moderately lower as compared to T3/T4, suggesting that PPARG might play an important role in tumorigenesis of BRCA at the very early stage. In the advanced stages, the DNA methylation level of PPARG was increased in N2/N3 compared with N0/N1, supporting the DNA methylation might result in the declining of PPARG expression in metastatic development.

Next, we investigated the relationship between PPARG and the tumor immune microenvironment. We observed the significant correlation between immune score and better prognosis, and the positive correlation between the expression level of PPARG with the immune score. CIBERSORT algorithm analysis exhibited that PPARG has a positive correlation with B cells naive and T cells CD4 memory resting. Tumor immunotherapy has greatly changed the prognosis of cancer patients. Immune checkpoint blockade therapy has been shown to improve the survival rate of patients with advanced melanoma, non-small cell lung cancer, and other cancers. Therefore, for future possibilities we tried to assess the relationship between PPARG and immune checkpoint. Through the TIMER database, we found that there is a positive correlation between PPARG and most immune checkpoints (CD160, CD96, IL10RB, IL10, CTLA4, PDCD1LG2, PDCD1, CSF1R, CD274, and CD244). Further, TCGA patients were divided into high group and low group according to the expression level of PPARG. The outcomes revealed that the high PPARG expression group had a higher level of immune checkpoint expression. These findings support PPARG as an additional biomarker for prognosis after immunotherapy and an additional target to facilitate corresponding immunotherapy (Ding et al., 2020; Ma et al., 2021). However, in-depth studies are required to establish further facts.

In addition, we identified the degree of tumor mutational burden of BCRA and examined the relationship between PPARG and TMB. The outcomes depicted that patients with lower PPARG had higher TMB levels. The low expression of PPARG in the tumor and declining of its anti-tumor function could be due to the BRCA development and TMB accumulation. Finally, we divided TCGA patients into high and low PPARG groups according to the expression level of PPARG and analyzed the main enrichment pathways through GSEA software.

Recent studies confirmed that PPARG activation facilitates the anti-tumor effect of 6-iodolactone (Nava-Villalba et al., 2015) and hesperetin (Hermawan et al., 2021). According to a study carried by Liu stated that datasets of GEO revealed that the high expression of PPARG was associated with better prognosis in BRCA (Liu et al., 2017). In addition, some researchers mentioned that PPARG can inhibit tumor progression (Xu et al., 2019; Lian et al., 2020; Shi et al., 2020). These studies are in accordance with our data that patients with high expression of PPARG are likely to have a better prognosis. However, most of the existing studies on PPARG in breast cancer are one-sided, focusing only on a single research field. In this study, we thoroughly evaluated the prognosis of PPARG in BRCA and its specific characteristics, including DNA methylation, tumor immune microenvironment, and tumor mutation burden. These results confirmed the importance of PPARG expression in cancer prognosis and treatment and identified important areas for further exploration and confirmation.

## Conclusion

Findings of our study identified the PPARG as a potential biomarker by confirming its low expression in BRCA and its correlation to prognosis. Moreover, its correlation to DNA methylation and tumor microenvironment may guide new therapeutic strategies for BRCA patients.

## Data Availability Statement

The original contributions presented in the study are included in the article/Supplementary Material, further inquiries can be directed to the corresponding authors.

## Author Contributions

XH and LL designed the study, analyzed the data, and wrote the manuscript. XH, LL, ZC, and ML analyzed the data and contributed in writing the manuscript. JW performed the experiments, analyzed the data, and wrote the manuscript. All authors contributed to the article and approved the submitted version.

## Conflict of Interest

The authors declare that the research was conducted in the absence of any commercial or financial relationships that could be construed as a potential conflict of interest.

## Publisher’s Note

All claims expressed in this article are solely those of the authors and do not necessarily represent those of their affiliated organizations, or those of the publisher, the editors and the reviewers. Any product that may be evaluated in this article, or claim that may be made by its manufacturer, is not guaranteed or endorsed by the publisher.

## Footnotes

1
https://portal.gdc.cancer.gov

2
https://www.ncbi.nlm.nih.gov/geo/query/acc.cgi?acc=GSE42568

3
https://string-db.org/

4
http://cis.hku.hk/TISIDB/index.php

5
https://cistrome.shinyapps.io/timer/

6
http://ualcan.path.uab.edu

7
http://kmplot.com/analysis/

## Supplementary Material

Supplementary Figure 1 The Kaplan-Meier plotter of PPARG.

Supplementary Figure 2 GSEA enrichment analysis.

Supplementary Table 1 Clinical information.

## Abbreviations

BRCA: Breast cancer
PPI: Protein-Protein Interaction
PPARG: Peroxisome Proliferator Activated Receptor Gamma
RXRs: retinoid X receptors
TZDs: thiazolidinediones
RNA-Seq: RNA sequencing
WGCNA: weighted gene co-expression network analysis
GO: gene ontology
KEGG: Kyoto Encyclopedia of Genes and Genomes
DEG: differential gene.
