# Supplementary figures and images for: Integrated Analysis of the Expression Characteristics, Prognostic Value, and Immune Characteristics of PPARG in Breast Cancer
Source: Front Genet. 2021 Sep 9;12:737656. doi: 10.3389/fgene.2021.737656 (PMC8458894; doi:10.3389/fgene.2021.737656)

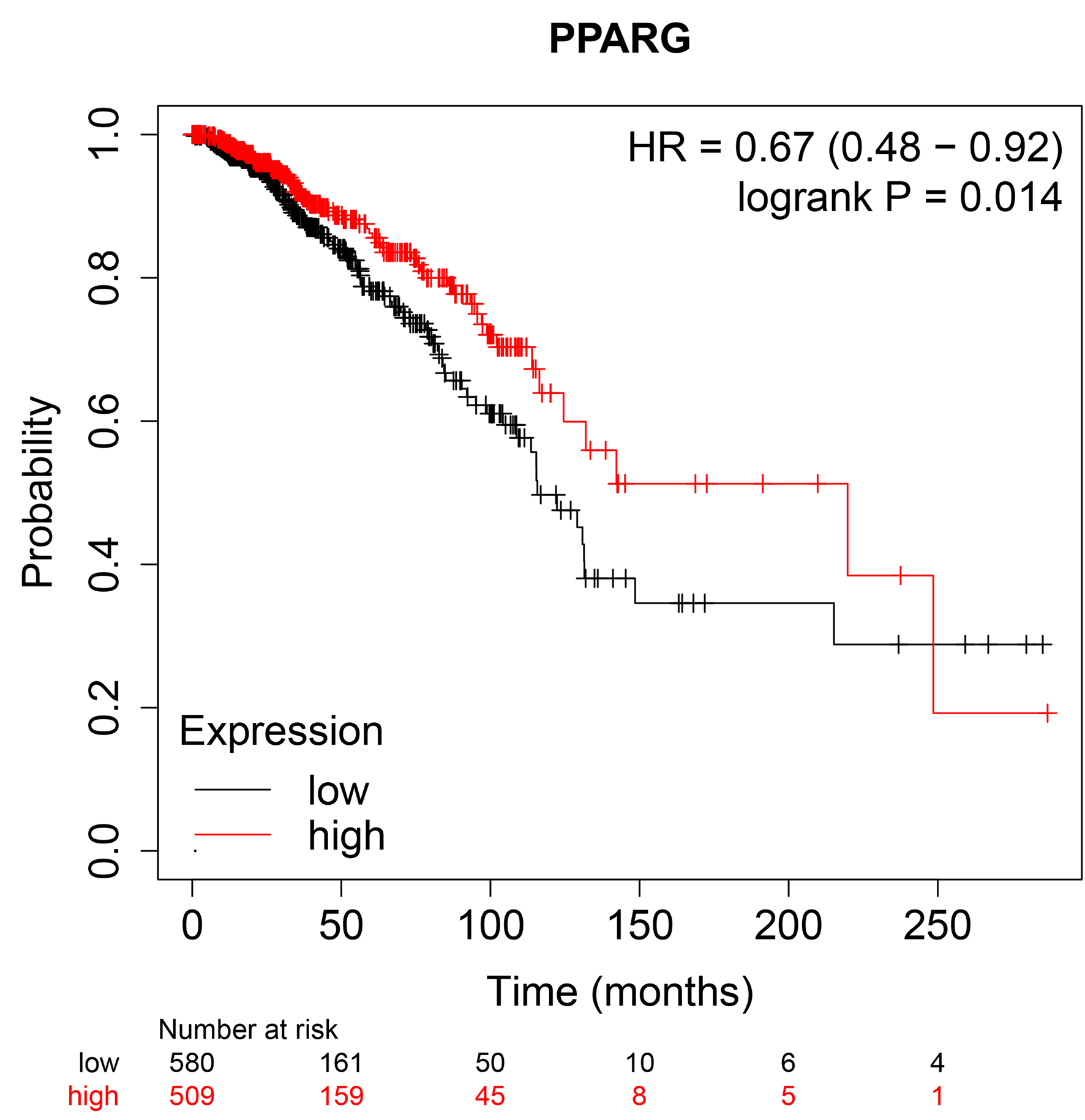

Supplement: Supplementary Figure 1 — The Kaplan-Meier plotter of PPARG. [file Image_1.TIF]

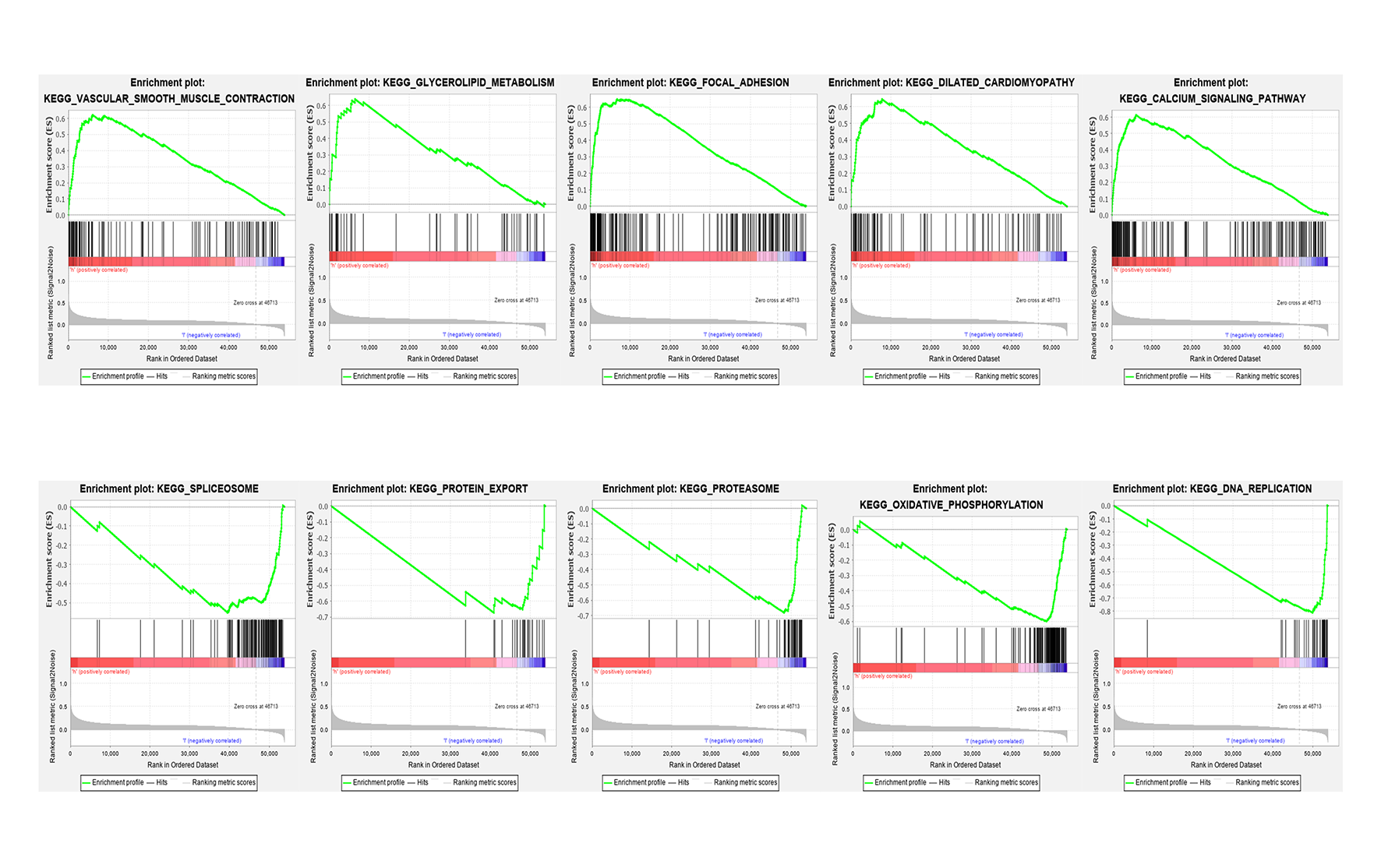

Supplement: Supplementary Figure 2 — GSEA enrichment analysis. [file Image_2.TIF]
