# Supplementary material for: Integrated Analysis of the Expression Characteristics, Prognostic Value, and Immune Characteristics of PPARG in Breast Cancer
Source: Front Genet. 2021 Sep 9;12:737656. doi: 10.3389/fgene.2021.737656 (PMC8458894; doi:10.3389/fgene.2021.737656)
Supplement: Supplementary Table 1 — Clinical information. [file Table_1.docx]

**TABLE S1**

**clinical information.**

|  |  | PPARG expression | | | | PPARG methylation | | | |
| --- | --- | --- | --- | --- | --- | --- | --- | --- | --- |
| Covariates | Type | Total | High | Low | Pvalue | Total | High | Low | Pvalue |
| Age | <=65 | 566(72.38%) | 288(73.66%) | 278(71.1%) | 0.4716 | 566(72.38%) | 283(72.38%) | 283(72.38%) | 1 |
| Age | >65 | 216(27.62%) | 103(26.34%) | 113(28.9%) |  | 216(27.62%) | 108(27.62%) | 108(27.62%) |  |
| M | M0 | 614(78.52%) | 305(78.01%) | 309(79.03%) | 0.9861 | 614(78.52%) | 305(78.01%) | 309(79.03%) | 0.5712 |
| M | M1 | 13(1.66%) | 7(1.79%) | 6(1.53%) |  | 13(1.66%) | 8(2.05%) | 5(1.28%) |  |
| M | unknow | 155(19.82%) | 79(20.2%) | 76(19.44%) |  | 155(19.82%) | 78(19.95%) | 77(19.69%) |  |
| N | N0 | 348(44.5%) | 168(42.97%) | 180(46.04%) | 0.1343 | 348(44.5%) | 166(42.46%) | 182(46.55%) | 0.1486 |
| N | N1 | 270(34.53%) | 136(34.78%) | 134(34.27%) |  | 270(34.53%) | 130(33.25%) | 140(35.81%) |  |
| N | N2 | 95(12.15%) | 46(11.76%) | 49(12.53%) |  | 95(12.15%) | 57(14.58%) | 38(9.72%) |  |
| N | N3 | 57(7.29%) | 37(9.46%) | 20(5.12%) |  | 57(7.29%) | 31(7.93%) | 26(6.65%) |  |
| N | unknow | 12(1.53%) | 4(1.02%) | 8(2.05%) |  | 12(1.53%) | 7(1.79%) | 5(1.28%) |  |
| T | T1 | 196(25.06%) | 108(27.62%) | 88(22.51%) | 1.00E-04 | 196(25.06%) | 83(21.23%) | 113(28.9%) | 0.0448 |
| T | T2 | 450(57.54%) | 204(52.17%) | 246(62.92%) |  | 450(57.54%) | 234(59.85%) | 216(55.24%) |  |
| T | T3 | 109(13.94%) | 72(18.41%) | 37(9.46%) |  | 109(13.94%) | 56(14.32%) | 53(13.55%) |  |
| T | T4 | 24(3.07%) | 7(1.79%) | 17(4.35%) |  | 24(3.07%) | 16(4.09%) | 8(2.05%) |  |
| T | unknow | 3(0.38%) | 0(0%) | 3(0.77%) |  | 3(0.38%) | 2(0.51%) | 1(0.26%) |  |
| Gender | female | 773(98.85%) | 388(99.23%) | 385(98.47%) | 0.5025 | 773(98.85%) | 386(98.72%) | 387(98.98%) | 1 |
| Gender | male | 9(1.15%) | 3(0.77%) | 6(1.53%) |  | 9(1.15%) | 5(1.28%) | 4(1.02%) |  |
| expression | High | 391(50%) | 391(100%) | 0(0%) | 0 | 391(50%) | 152(38.87%) | 239(61.13%) | 0 |
| expression | Low | 391(50%) | 0(0%) | 391(100%) |  | 391(50%) | 239(61.13%) | 152(38.87%) |  |
| methylation | High | 391(50%) | 152(38.87%) | 239(61.13%) | 0 | 391(50%) | 391(100%) | 0(0%) | 0 |
| methylation | Low | 391(50%) | 239(61.13%) | 152(38.87%) |  | 391(50%) | 0(0%) | 391(100%) |  |
